# Supplementary material for: SFPQ and NONO suppress RNA:DNA-hybrid-related telomere instability
Source: Nat Commun. 2019 Mar 1;10:1001. doi: 10.1038/s41467-019-08863-1 (PMC6397292; doi:10.1038/s41467-019-08863-1)
Supplement: Supplementary file 1 — Supplementary Information [file 41467_2019_8863_MOESM1_ESM.pdf]

SUPPLEMENTARY INFORMATION

SFPQ and NONO suppress RNA:DNA hybrid related telomere instability

Petti et al.

Supplementary Methods:

Cell lines and cell culture

Feeder-independent mouse ESCs were cultured on 0.2% gelatin-coated plates in mESC self-renewal medium (Dulbecco's modified Eagle's medium (DMEM) supplemented with 15% knockout serum replacement (Gibco), 1% nonessential amino acids (Gibco), 1mM sodium pyruvate (Gibco), 1% L-glutamine (Gibco), 0.1 mM β-mercaptoethanol, 1% penicillin/streptomycin (Gibco) and 1000U/ml mouse Leukemia Inhibitory Factor (LIF). Human cancer cell lines were obtained from ATCC and have not been cultured for longer than 6 months. H1299 (carcinoma; non–small cell lung cancer) cells were cultured in Roswell Park Memorial Institute (RPMI) medium (Lonza) supplemented with 10% fetal bovine serum (Gibco), 1% L-glutamine (Gibco), 1% penicillin/streptomycin (Gibco). U-2 OS (osteosarcoma) cells were cultured in low glucose Dulbecco's modified Eagle's (DMEM) medium (Lonza) with 10% fetal bovine serum (Gibco), 1% L-glutamine (Gibco), 1% penicillin/streptomycin (Gibco). H1299 and U-2 OS cells transduced with the retroviral vector pLPC or pLPC-FLAGp54nrb vectors were selected with puromycin (1□g/ml). H1299 and U-2 OS cells transfected with linearized pLPC (control) vector or with a combination of linearized pLPC and pCMVmyc-SFPQ-WT (addgene) vectors were selected using puromycin (1□g/ml). Treatments: hydroxyurea (HU) (Sigma), 5 mM for 6 hours; 5-Aza-2'deoxycytidine (Sigma) 10µM for 72 hours.

TERRA RNA-pull down

Cells were re-suspendend in Buffer A (10 mM HEPES pH 7.9, 1.5 mM $MgCl_2$, 10 mM KCl, 0.5 mM DTT, 1 mM PMSF and complete proteinase inhibitor (Roche)), incubated on ice for 10 minutes and homogenized in the presence of 0.05% NP-40 using a Dounce homogenizer pestle. Obtained nuclei were pelleted and re-suspended in Buffer C* (20 mM HEPES pH 7.9, 25% (v/v) glycerol, 1.5 mM $MgCl_2$, 0.2 mM EDTA, 1 mM PMSF, 0.5 mM DTT, complete protease inhibitor (Roche)). NaCl was added to reach a final concentration of 400mM. Extracts were incubated on ice for 20 minutes and sonicated. Samples were centrifuged to obtain the supernatant containing nuclear proteins. NaCl concentration was diluted with Buffer C diluent (20 mM HEPES pH7.9, 1.5 mM $MgCl_2$, 0.2 mM EDTA, 0.5 mM DTT, 1 mM PMSF, complete protease inhibitor (Roche)) to reach a final concentration of 150mM NaCl. Extracts were pre-cleared using streptavidin agarose beads (Invitrogen). Beads were removed by centrifugation and nuclear extracts were supplied with RNase Inhibitor (RNase

OUT, Invitrogen, final concentration 300U/ml), yeast tRNA (Invitrogen, final concentration 100ng/ml), Heparin (Sigma, final concentration 5ug/ml), RNA oligos biotin-r[UUAGGG]$_6$ (5' UUAGGGUUAGGGUUAGGGUUAGGGUUAGGGUUAGGG 3') or biotin-r[EGFP] (5' AAGGACGACGGCAACUACAAGACCCGCGCCGAGGUGAAGU 3') (eurofins) and streptavidin agarose beads (Invitrogen). Samples were incubated for 1hr at 4°C. Beads were washed in Binding Buffer (20 mM HEPES pH 7.9, 20% glycerol, 0.2 mM EDTA, 150 mM NaCl, 0.05% NP-40, 1mM PMSF, 0.5 mM DTT, complete protease inhibitor cocktail (Roche), 300U/ml RNase OUT (Invitrogen)) and bound proteins were eluted using Laemmli sample buffer (2X). Eluted proteins were separated on a one dimensional SDS-PAGE and subjected to silver staining (Invitrogen). Specific biotin-r[UUAGGG]$_6$ binding proteins were cut out from the gel and identified by mass spectroscopy.

MALDI-MS and MALDI-MS/MS analysis

Gels were stained using a mass spectrometry-compatible silver staining (SilverQuest™ Kit, Invitrogen). Selected lanes were excised from the gels, de-stained and washed twice in 50% ACN with 50 mM ammonium bicarbonate, dehydrated in 100% ACN. In-gel reduction was performed by incubating lanes in a 10 mM DTT, 100 mM ammonium bicarbonate solution for 30 min at 56°C and alkylation in 55 mM iodoacetamide, 100 mM ammonium bicarbonate for 20 min at room temperature. Protein samples were dehydrated again in 100% ACN and digested by rehydrating gel pieces in 50 mM ammonium bicarbonate containing 4 ng/uL of trypsin (#V5111, Promega) overnight at 37°C. Tryptic peptides were desalted and concentrated using ZipTip mC18 pipet tips (Millipore) and co-eluted onto the MALDI target in 1 µL of α-cyano-4-hydroxycinnamic acid matrix (5 mg/mL in 50% ACN,0.1% TFA).

Mass spectra were acquired over a mass range of 800–4000 m/z (Nd:YAG laser at 355 nm, 40 Shots/Sub-Spectrum for 2,000 Total Shots/Spectrum) by reflectron positive mode on an Applied Biosystems 4,800 Proteomics Analyzer mass spectrometer (Applied Biosystems) and calibrated using a standard mixture (Mass Standards Kit, AB SCIEX). MS/MS spectra were acquired in positive mode (Nd:YAG laser at 355 nm, 40 Shots/Sub-Spectrum for 4,000 Total Shots/Spectrum) and MS/MS calibration was achieved by using the default calibration method.

Protein identifications were performed with the ProteinPilotTM software (version 2.0.1; Applied Biosystems) using the Paragon^{TM} algorithm as the search engine. Each MS/MS spectrum was searched against an Uniprot/SwissProt database of mouse. The search parameters allowed for cysteine modification by iodoacetamide and biological modifications

programmed in the algorithm (i.e., phosphorylations, semitryptic fragments, etc.). The detected protein threshold (ProtScore) in the software was set to 1.3 to achieve 95% confidence interval.

siRNAs and plasmids transient transfection

For siRNAs transfection RNAi-MAX Lipofectamine (Invitrogen) was used according to the manufacturer's suggestions. siRNAs listed in Supplementary Table 1 have been transfected at a final concentration of 30 nM for 72 hours. For plasmids transfections Lipofectamine 2000 (Invitrogen) was used according to the manufacturer's suggestions. One microgram of the following plasmids has been transfected for 72 hours: pcDNA 3.1-mCherry (provided by Alessandro Marcello, ICGEB, Trieste), pLHCX-mCherry-RNase H1 (provided by Fabrizio D'adda di Fagagna, IFOM, Milan).

Western Blot

Whole-cell lysates and nuclear extracts were prepared as previously described [1]. Protein extracts were subjected to Western blotting according to standard procedures. Originals of representative images are shown as Supplementary figures 6 and 8. Primary antibodies are listed in Supplementary table 2.

ChIP assay and telomere dot-blots

Chromatin immunoprecipitation of telomeric and subtelomeric chromatin was performed as described [2]. Formaldehyde fixed chromatin of $2x10^7$ cells was immunoprecipitated using 3.5 µg of purified antibodies (**Supplementary table 2**) or rabbit/mouse control IgGs. DNA was purified from immunoprecipitated chromatin by RNA and Proteinase K digestion, phenol-chloroform extraction; followed by ethanol precipitation. DNA was transferred to a nitrocellulose membrane and hybridized with a dCTP-[α-^{32}P]-labeled telomeric probe (gift from Maria Blasco, CNIO, Madrid; Random primers DNA labeling system, Invitrogen). After stripping (3x 10 minutes in 0.5% SDS, 0.5x TE at 90°C), the membrane was hybridized with a dCTP-[α-^{32}P]-labeled AluY probe (pAluYNF1 plasmid, Addgene 50933)[3]. Originals of representative images are shown as Supplementary figure 7.

Microscopy

Cells subjected to immunofluorescence, RNA-FISH, immunofluorescence combined with RNA-FISH, CO-FISH or telomere DNA FISH were analyzed using a Leica DM4000B

microscope equipped with a Leica DFC420C digital camera. Images were captured using Leica Application Suite (LAS) imaging software. For confocal analysis of immunostained cells a Nikon Eclipse C1si confocal microscope system was used. Images were processed by using ImageJ 1.46r (NIH, Bethesda, USA). In all experiments single focal planes were used for analysis. Only for Fig. 3H, three focal planes were used for analysis

Immunoprecipitation

Cell lysates were prepared with ice-cold lysis buffer containing 50 mM Tris-HCl, pH 8, 150 mM NaCl, 5mM EDTA, 1mM DTT, 1mM PMSF, 1 mM beta-glycerophosphate, 1mM Na_3VO_4, 5 mM Na_2F, 5% glycerol, 1% NP-40 and protease inhibitors cocktail (Sigma). After centrifugation and pre-clearing with protein-A agarose-beads (Santa Cruz) for 30 minutes, lysates were incubated at 4 °C with specific antibodies (**Supplementary table 2**) or rabbit IgGs as negative control. After 2 hours, protein-A agarose-beads (Santa Cruz) were added to each IP and incubated overnight. After washing, bound proteins were eluted in Laemmli buffer (2x) and subjected to SDS-PAGE followed by western blotting.

Immunofluorescence combined with telomere DNA FISH on metaphase spreads

H1299 cells were blocked in metaphase using colcemid (1ug/ml for 3 hours), collected by trypsinization and resuspended in hypotonic buffer (0.2% (w/v) KCl, 0.2% (w/v) trisodium citrate) at room temperature (RT) for 10 minutes. Resuspendend cells were cytocentrifuged on slides pre-treated with 50 µg/ml Poly-L-Lysin (Sigma). Cells were fixed for 20 minutes with 4% formaldehyde (1XPBS) and permealized with PBS 0.1% Triton X-100 for 7 minutes at room temperature. Cells were blocked with 3% (w/v) BSA (1XPBS) for 20 minutes at 37°C. Subsequently, slides were incubated with primary antibodies (**Supplementary table 2**) diluted in blocking solution at 4° C overnight. Slides were washed twice in 0.05% Triton X-100, 1xPBS for 5 minutes and then incubated with secondary antibodies (**Supplementary table 3**) for 1 hour at room temperature. Alter incubation with secondary antibodies, cells were fixed in 4% formaldehyde (1XPBS) and subjected to standard telomere DNA FISH.

Quantitative TERRA RT-PCR

TERRA RT-PCR analysis was carried out as previously described [4]. Total RNA (1.5 µg) was treated with DNase RQ1 (Promega) for 30 minutes at 37°C and reverse transcribed with random oligos and telomere-specific primers $(CCCTAA)_5$ (**Supplementary table 4**,) at 55 °C using SuperScript III reverse transcriptase (Invitrogen). Controls without reverse

transcriptase included in subsequent steps. Expression levels of different TERRA transcripts primed from distinct subtelomeric primers were determined by SYBR green realt-time PCR (Applied Biosystems) using chromosome specific primers (Chr. 1q-21q and Chr. 2q-10q-13q; **Supplementary table 4).** Expression levels were normalized to histone H3 mRNA levels (primers listed in **Supplementary table 4**).

TERRA Northern Blot

Total RNA was prepared using QIAzol Lysis Reagent (Qiagen) according to the manufacturer's instructions. To perform Northern blot analysis 10 µg of total RNA was loaded onto a 1.2% agarose gel containing formaldehyde and separated by electrophoresis as previously described [5]. RNA was then transferred overnight to a Nylon membrane and hybridized with a ^{32}P-labelled TERRA probe. A GAPDH probe was used to control of equal loading of RNA.

Supplementary References:

1. Dinami, R. *et al.* miR-155 drives telomere fragility in human breast cancer by targeting TRF1. *Cancer Res.* **74,** 4145–56 (2014).
2. García-Cao, M., O'Sullivan, R., Peters, A. H. F. M., Jenuwein, T. & Blasco, M. A. Epigenetic regulation of telomere length in mammalian cells by the Suv39h1 and Suv39h2 histone methyltransferases. *Nat. Genet.* **36,** 94–9 (2004).
3. Wallace, M. R. *et al.* A de novo Alu insertion results in neurofibromatosis type 1. *Nature* **353,** 864–866 (1991).
4. Arnoult, N., Van Beneden, A. & Decottignies, A. Telomere length regulates TERRA levels through increased trimethylation of telomeric H3K9 and HP1α. *Nat. Struct. Mol. Biol.* **19,** 948–56 (2012).
5. Schoeftner, S. & Blasco, M. A. Developmentally regulated transcription of mammalian telomeres by DNA-dependent RNA polymerase II. *Nat. Cell Biol.* **10,** 228–36 (2008).

Supplementary Table 1: siRNAs

Target	Company	Cod/sequence
**human NONO**	Dharmacon	ON-TARGETplus smartpool (L-007756-01)
**human RNAseH1**	Eurofins	5’ ACAAACCAAAGAGCGGAAAUUCAUG(dTdT) 3’ (Arora R. et al. 2014)
**human SFPQ**	Dharmacon	ON-TARGETplus smartpool (L-006455-00)
**human TERF1**	Dharmacon	ON-TARGETplus smartpool (L-010542-00)
**Control-scamble**	Dharmacon	ON-TARGETplus Non-targeting siRNA #1 (D-001810-01-05)

Supplementary Table 2: primary antibodies

Antibodies	Company	WB	IF	IP	Chip
**Mouse anti–Actin**	Sigma, A2228	1:10000			
**Mouse anti-Myc-tag (9B11)**	Cell Signaling, 2276	1:1000			
**Mouse anti-Flag-tag M2 (clone M2)**	Sigma, F3165	1:1000			
**Mouse anti-DNA-RNA Hybrid (S9.6)**	Kerafast, ENH001		1:150		
**Mouse anti-FUS/TSL (4H11)**	Santa Cruz, sc-47711	1:1000			
**Mouse anti- γH2AX (ser139) (clone JBW301)**	Millipore, 06-70	1:2000	1:200		
**Rabbit anti-global histone H3**	Abcam, ab1791	1:10000			3.5ug
**Rabbit anti-NONO**	A300-582A, Bethyl lab	1:5000	1:200	2ug	3.5ug
**Mouse anti-p53 (DO-1)**	Santa Cruz, sc-126	1:1000			
**Rabbit PML (H-238)**	Santa Cruz, sc-5621		1:100		
**Rabbit anti-SFPQ/SFPQ**	A301-321A, Bethyl lab	1:5000		2ug	3.5ug
**Mouse anti-SFPQ (clone B92)**	Sigma, P2860	1:5000	1:150		
**Rabbit anti-phospho RPA32(S33)**	A300-246A, Bethyl Lab		1:800		
**Rabbit anti-TRF1(N-19)**	Santa Cruz, sc-6165-R		1:100		
**Mouse anti-TRF1 (TRF-78)**	Abcam, ab10579	1:1000			
**Mouse anti-TRF2 (4A794)**	Millipore, 05-521	1:500	1:200		3.5ug
**Rabbit anti-RAD51 (H-92)**	Santa Cruz, sc-8349		1:200		
**Rabbit anti-RNase H1 (N2C3)**	GeneTex, GTX117624		1:200		
**Normal mouse IgG**	Santa Cruz, sc-2025				3.5ug
**Normal rabbit IgG**	Santa Cruz, sc-2027				3.5ug

Supplementary Table 3: immunofluorescence secondary antibodies

Antibodies	Company
**Goat anti-rabbit Alexafluor 488**	Invitrogen
**Goat anti-mouse Alexafluor 488**	Invitrogen
**Goat anti-rabbit Alexafluor 555**	Invitrogen
**Goat anti-mouse Alexafluor 555**	Invitrogen
**Goat anti-mouse Alexafluor 647**	Invitrogen

Supplementary Table 4: TERRA qRT-PCR oligos

Oligo name	Sequence
**TEL Reverse transcription**	5’ CCTAACCCTAACCCTAACCCTAACCCTAA 3’
**Chr. 1q-21q Fw**	5’ TCTCGGTGCGCAGGATTCAGA 3’
**Chr. 1q-21q Rev**	5’ GTCACAGACCAGTTAGAATG 3’
**Chr. 2q-10q-13q Fw**	5’ GTCAGAGACCAGTTAGAACG 3’
**Chr. 2q-10q-13q Rev**	5’ GGTGCGCAGGATTCAGAGAG 3’
**H3 Fw**	5’ GTGAAGAAACCTCATCGTTACAGGCCTGGT 3’
**H3 Rev**	5’ CTGCAAAGCACCAATAGCTGCACTCTGGAA 3’

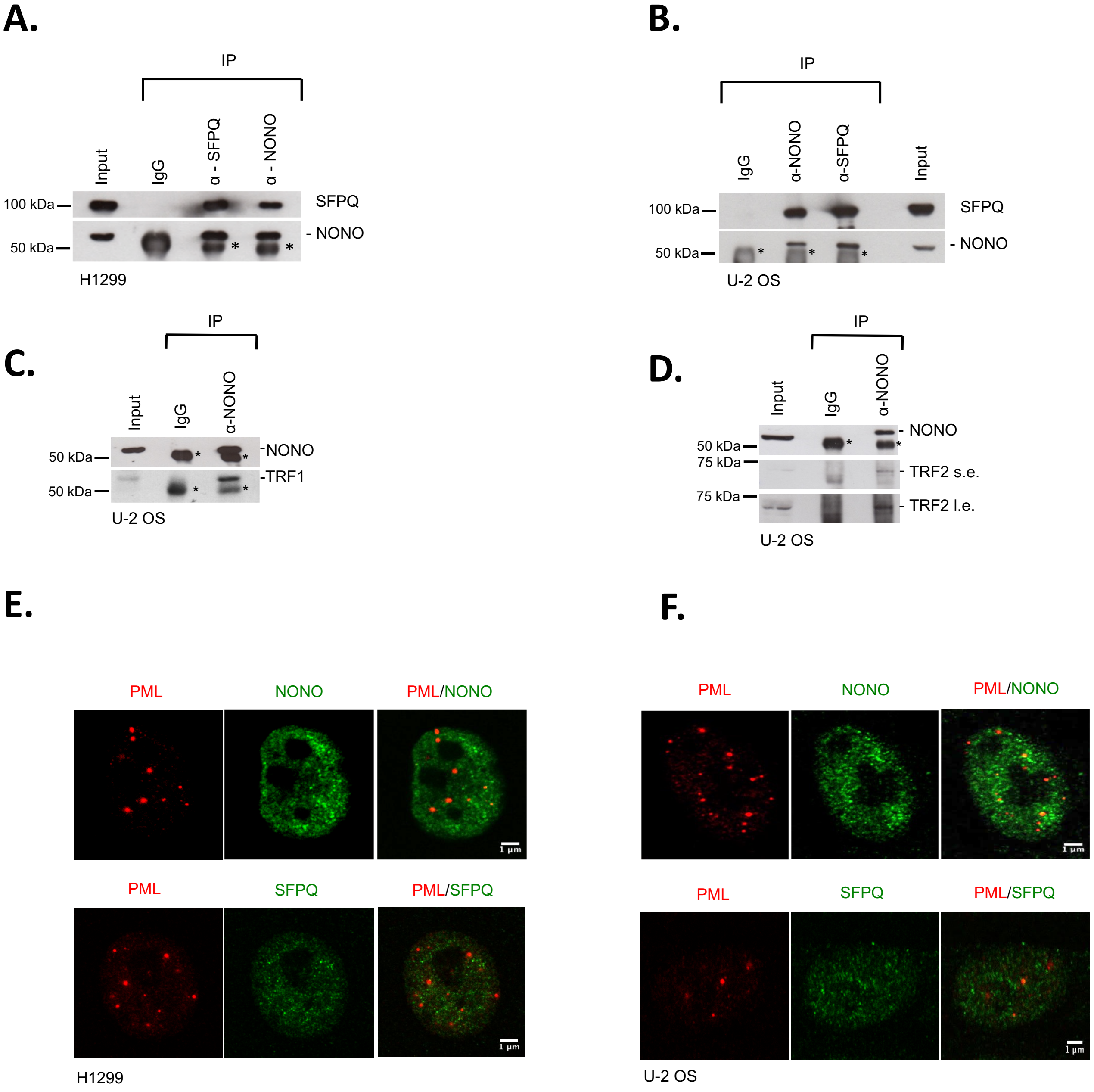

Supplementary Figure 1. **NONO and SFPQ interaction.**
A-B. Co-immunoprecipitation experiments with anti-SFPQ and anti-NONO antibodies in H1299 (A) and U-2 OS cells (B). Rabbit immunoglobulins were used as negative control. Asterisk indicates immunoglobulin heavy chain of antibodies used for immunoprecipitation. Horizontal short lines indicate the position of specific bands. **C,D:** Immunoprecipitation experiments using anti-NONO antibodies in U-2 OS cells. Immunoblots were probed with the indicated antibodies. Rabbit immunoglobulins were used as negative control. Asterisk indicates immunoglobulin heavy chain of antibodies used for immunoprecipitation. Horizontal short lines indicate the position of specific bands. s.e.: short exposure; l.e.: long exposure. **E. F.** Confocal microscope images showing the localization of PML, SFPQ and NONO in H1299 cells (E) and U-2 OS cells (F). Scale bar, 1 µm

Supplementary figure 1, related to Figure 1

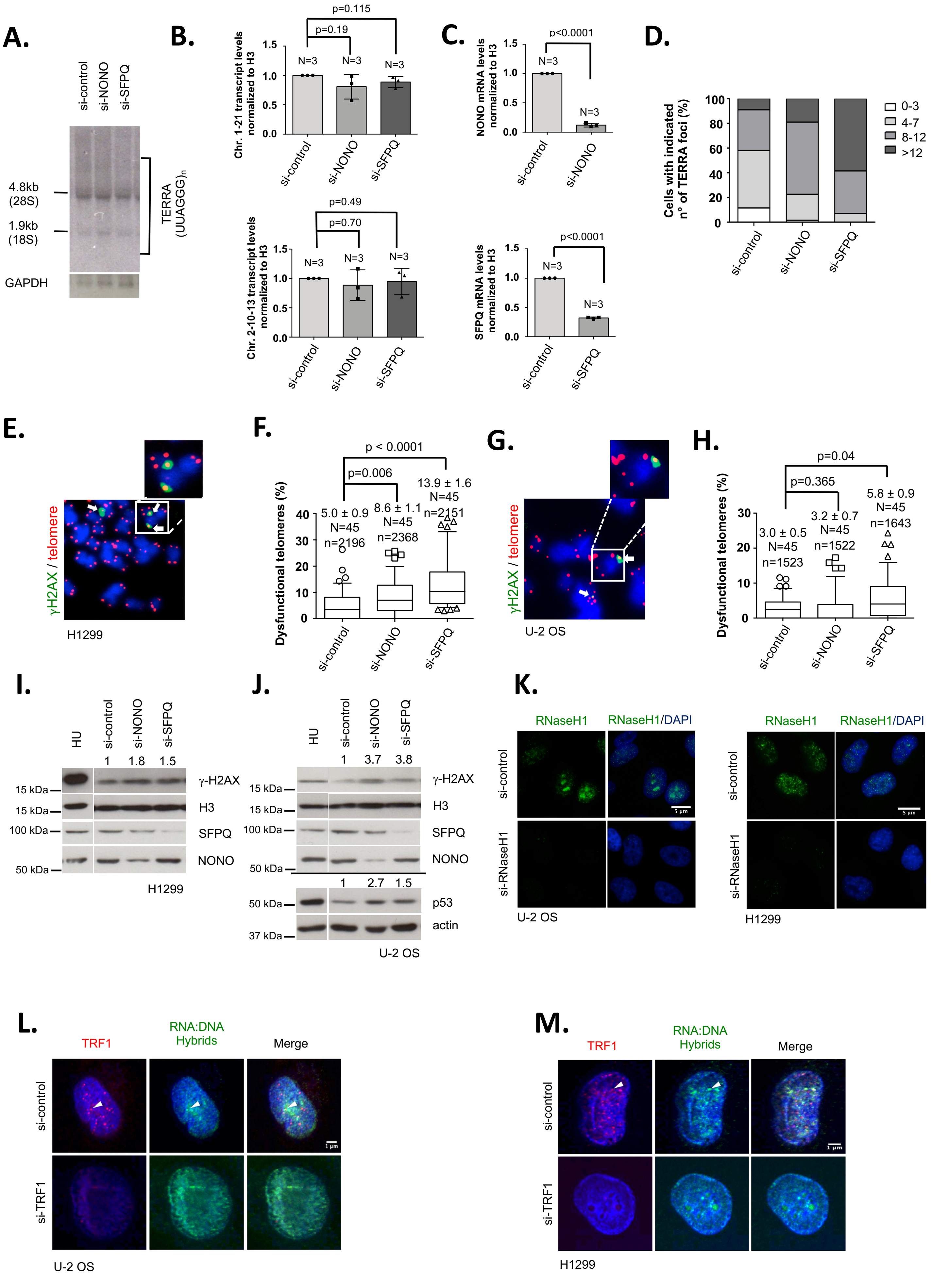

Supplementary Figure 2, related to figure 2

Supplementary Figure 2. **NONO and SFPQ do not impact on steady state TERRA expression levels in U-2 OS cells**.

A. Northern blotting of TERRA levels in U-2 OS cells transfected with indicated siRNAs. GAPDH probe was used as loading control. **B.** Quantification of TERRA transcripts from chromosomes 1-21 or chromosomes 2-10-13 by qRT-PCR in U-2 OS cells. Values were normalized against histone H3. **C.** NONO and SFPQ RNAi efficiency in U-2 OS cells of (B), by qRT-PCR. NONO and SFPQ mRNA levels were normalized against histone H3. **D.** TERRA foci number per nucleus in U-2 OS cells. **E, G.** Representative images of metaphase spreads stained with anti-γH2AX and subsequent telomeric DNA-FISH in H1299 (E) and U-2 OS (G) cells. **F, H.** Percentage of dysfunctional telomeres in H1299 (F) or U-2 OS cells (H). Box blot diagrams F-H: middle line represents median; boxes extend from the 25th to 75th percentiles. The whiskers mark the 10th and 90th percentiles. p values were calculated using a two-tailed Mann-Whitney test . Median telomere damage values and standard deviations are indicated; n=number of analyzed telomere repeat signals, N= number of analyzed metaphase spreads. **I, J.** Western blotting of nuclear extracts derived from H1299 (I) or U-2 OS (J) cells, transfected with indicated siRNAs. γH2AX levels were analyzed and normalized to histone H3. H1299 cells are mutant for p53. Values of quantitative densitometric analysis are shown. si-control was set to 1. HU, treatment with 5 mM hydroxyurea (HU) for 6 hours. **K.** Knock-down efficiency of RNaseH1 as determined by immunofluorescence. Scale bar, 5 µm **L, M.** Confocal immunofluorescence with anti-S9.6 and TRF1 antibodies in cells transfected with TRF1 or control siRNAs. Scale bar, 1 µm. **B, C.** si-control was set to 1. Means (bars) and standard deviation (error bars) are reported. N=number of independent experiments. A two-tailed Student's t-test was used to calculate statistical significance; p-values are shown. Source data is provided as supplementary information file.

Supplementary Figure 2, related to figure 2

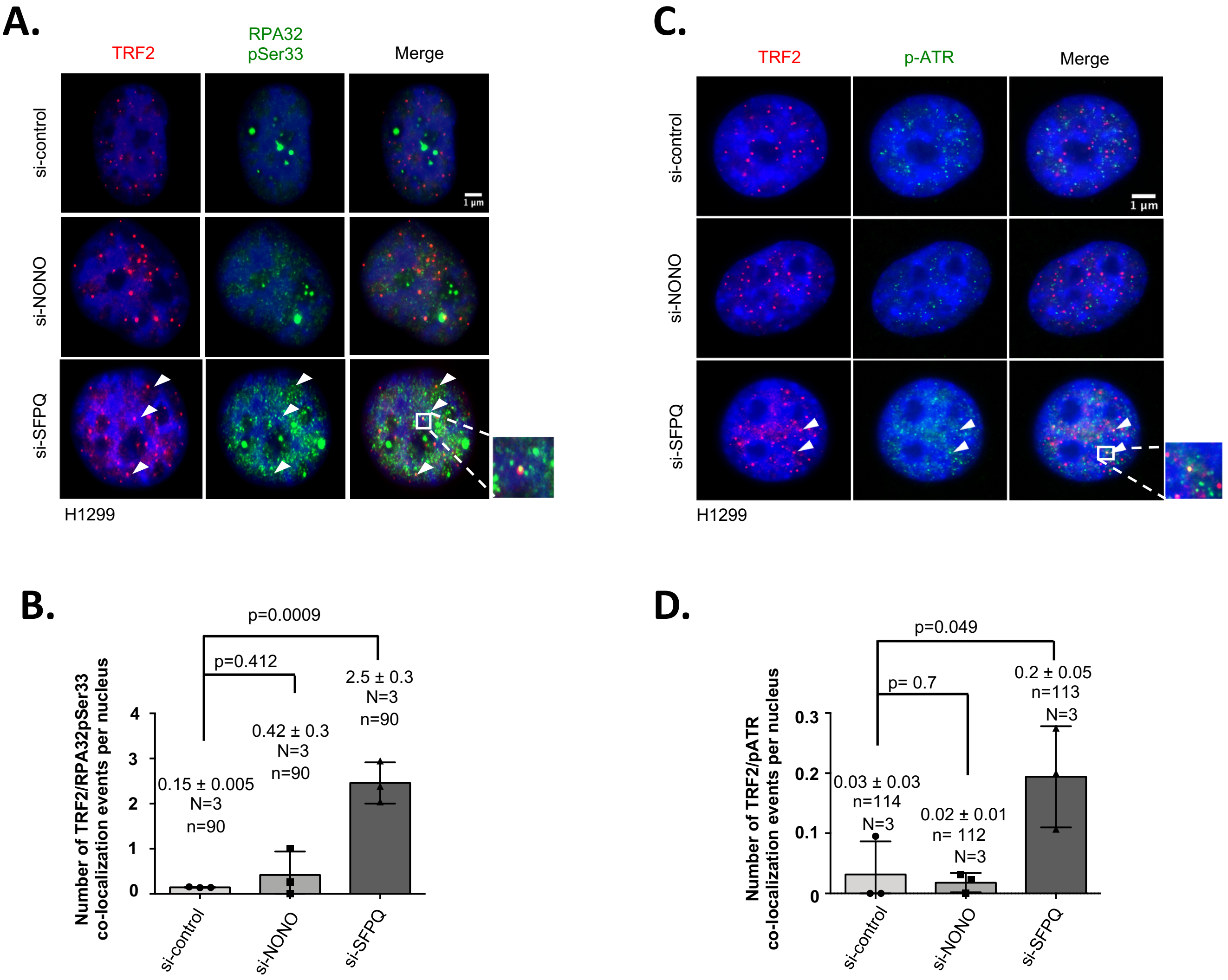

Supplementary Figure 3. Replication defects in NONO or SFPQ depleted H1299 cells.

A. Representative images of combined immunofluorescence with anti-TRF2 and anti-RPA32pSer33 antibodies in H1299 cells transfected with indicated siRNAs. **B.** Quantification of (A.), the number of TRF2/RPApSer33 co-localization events per nucleus is indicated. **C.** Representative images of combined immunofluorescence with anti-TRF2 and anti-pATR (S428) antibodies in H1299 cells transfected with indicated siRNAs. **D.** Quantification of (C.), the number of TRF2/pATR co-localization events per nucleus is indicated. B,D. N=number of independent experiments. n= number of analyzed nuclei. A two-tailed Student's t-test was used to calculate statistical significance; p-values are shown. Source data is provided as supplementary information file. Scale bar, 1 µm

Supplementary Figure 3, related to Figure 3

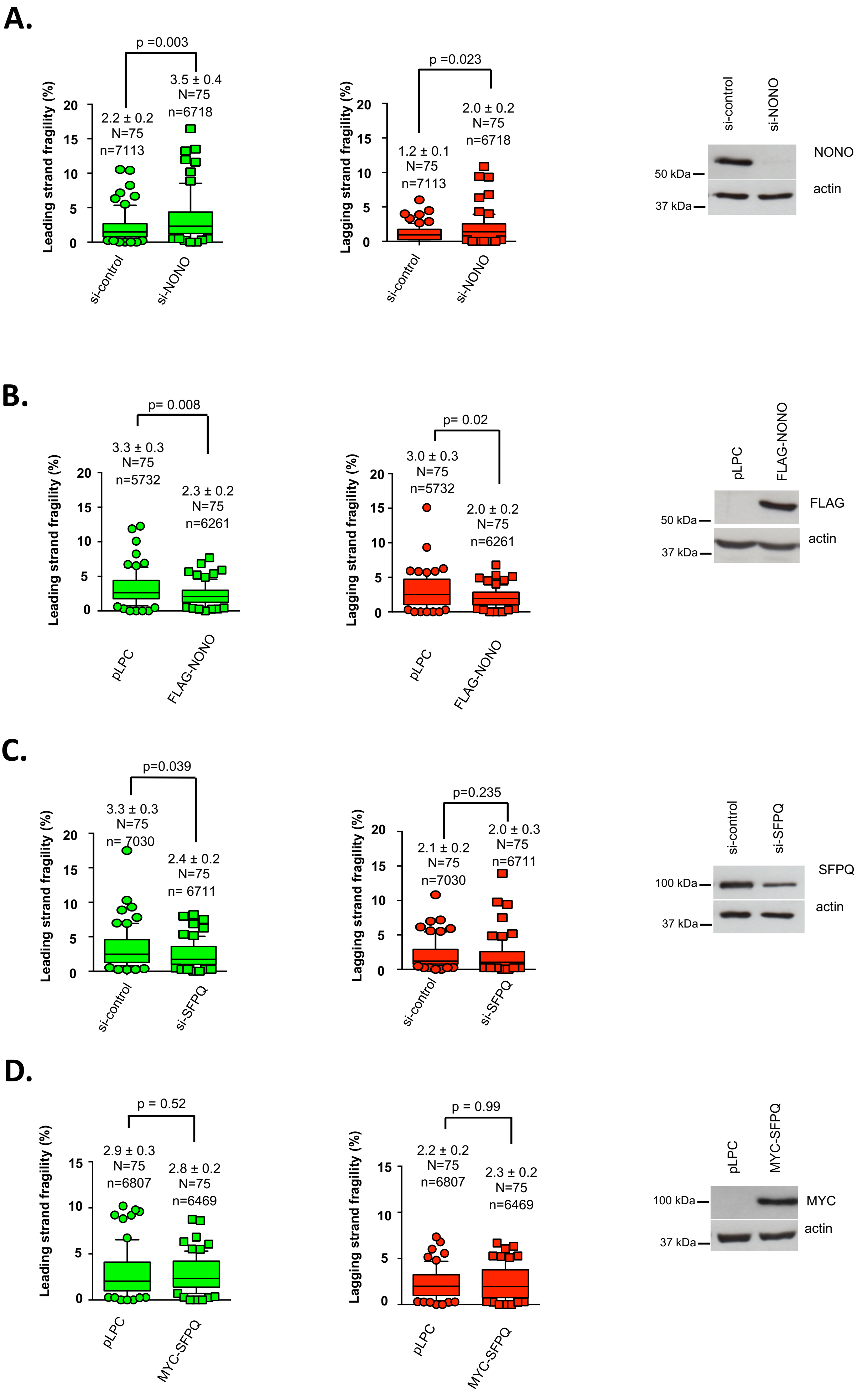

Supplementary figure 4, related to Figure 4

Supplementary Figure 4. NONO and SFPQ regulate telomere fragility of H1299 cells.

A. Quantification of leading strand fragility (left panel) and lagging strand fragility (central panel) in H1299 cells transfected with NONO specific siRNA or a non-targeting control siRNA. Right panel, western blotting experiments showing NONO knock-down efficiency. **B.** Quantification of leading strand fragility (left panel) and lagging strand fragility (central panel) in H1299 cells that stably overexpress flag-tagged NONO or the empty control vector. Right panel, western blotting experiments showing expression of flag-tagged NONO. **C.** Quantification of leading strand fragility (left panel) and lagging strand fragility (central panel) in H1299 cells transfected with SFPQ specific siRNA or a non-targeting control siRNA. Right panel, western blotting showing SFPQ silencing efficiency. **D.** Quantification of leading strand fragility (left panel) and lagging strand fragility (central panel) in H1299 cells that stably overexpress myc-tagged SFPQ or an empty control vector. Right panel, western blotting showing expression of myc-tagged SFPQ. In quantifications of panels A-D, each data point represents the fraction of fragile telomeres per number of telomeres in one analyzed metaphase. Only chromosome ends with detectable telomere sequences were considered for the quantification. Box blot diagrams A-D: middle line represents median; boxes extend from the 25th to 75th percentiles. The whiskers mark the 10th and 90th percentiles. p values were calculated using a two-tailed Mann-Whitney test . Median fragility values and standard deviation are indicated; n=number of analyzed telomere repeat signals, N= number of analyzed metaphase spreads.

Supplementary figure 4, related to Figure 4

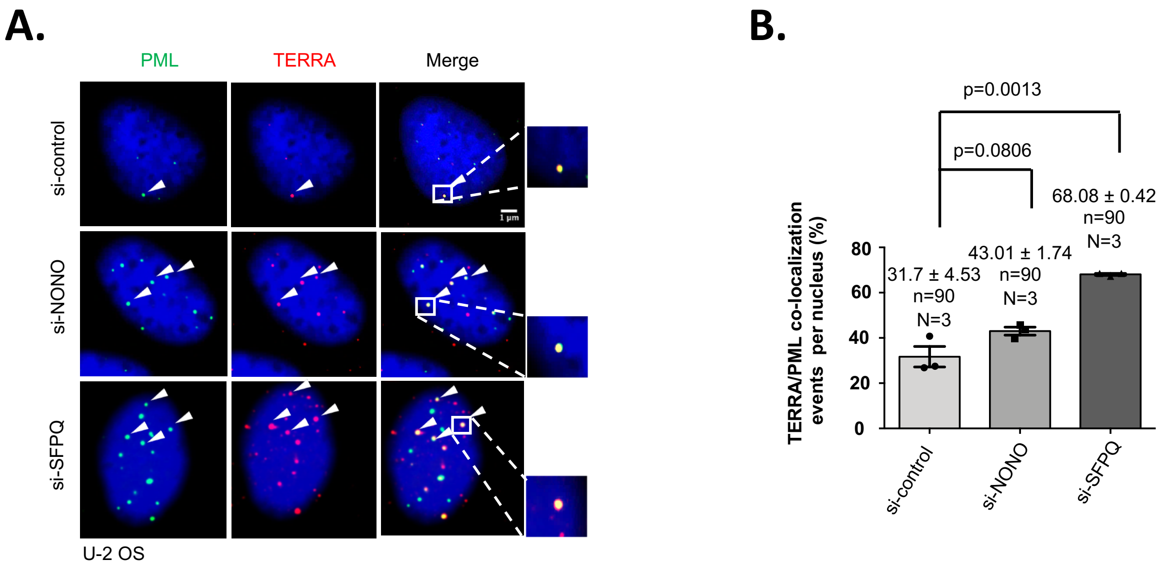

Supplementary Figure 5. Depletion of SFPQ does not induce APBs formation in H1299 cells.

A. Representative images of TERRA-PML co-staining in H1299 cells transfected with SFPQ specific siRNA or a non-targeting control siRNA. **B** Quantification of the number of TERRA-PML co-localizations per nucleus. Mean values (bars) and standard deviation (error bars) are reported. N=number of independent experiments. n= number of analyzed nuclei. A two-tailed Student's t-test was used to calculate statistical significance; p-values are shown. Source data is provided as supplementary information file. Scale bar, 1 μm

Supplementary figure 5, related to Figure 5

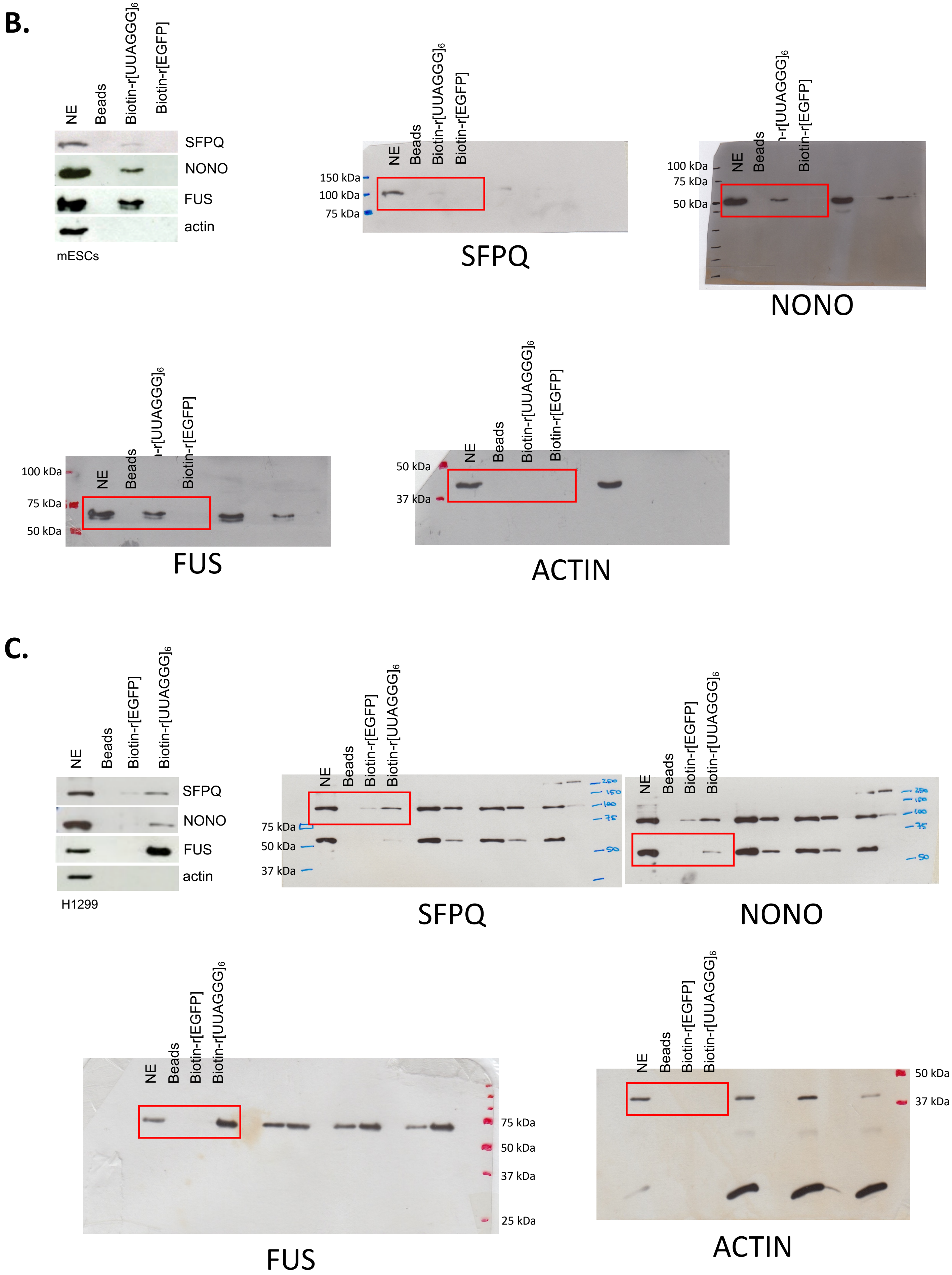

Supplementary Figure 6, referring to western blots in Figure 1

G.

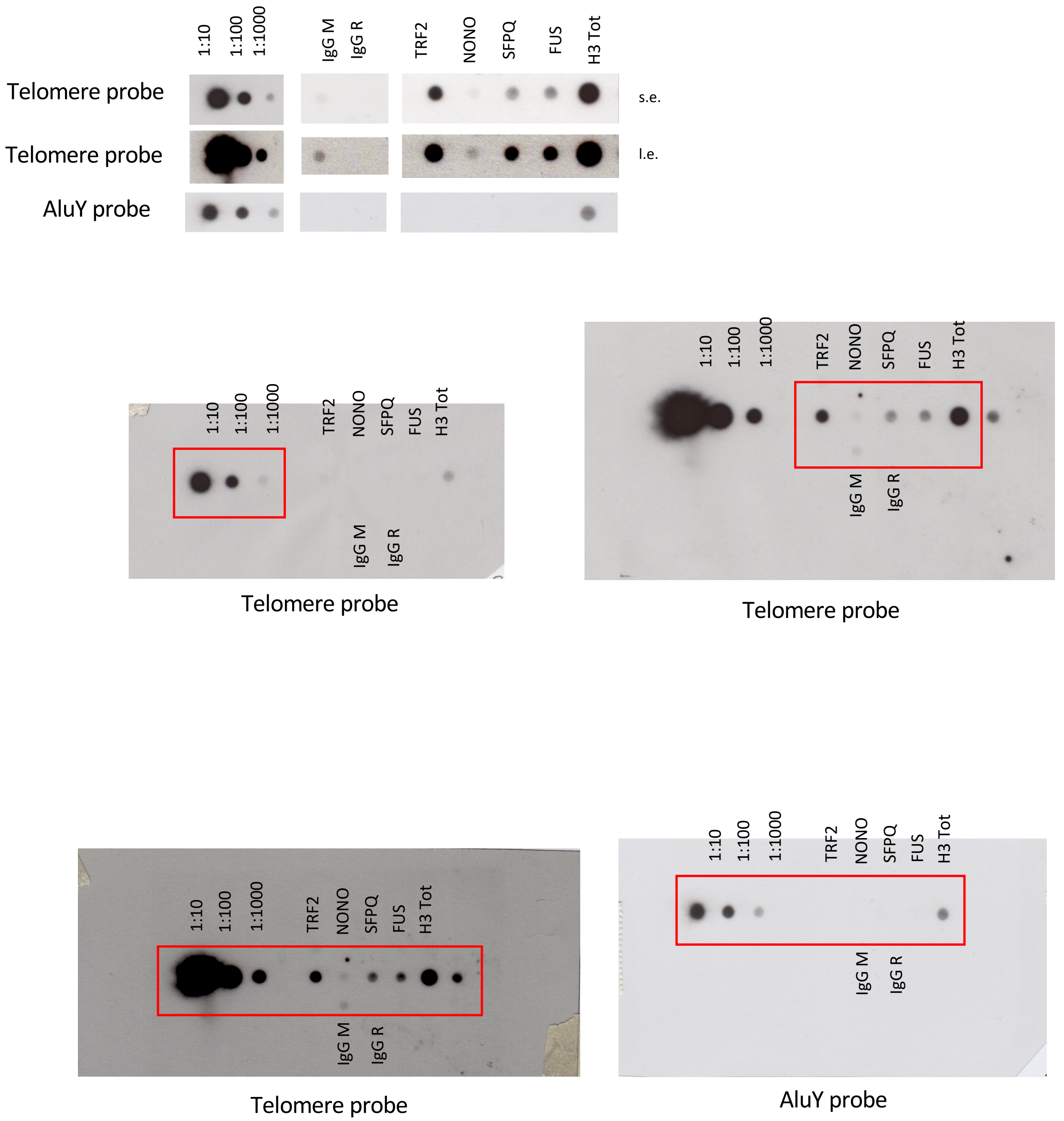

Supplementary Figure 7, referring to dot blots in Figure 1

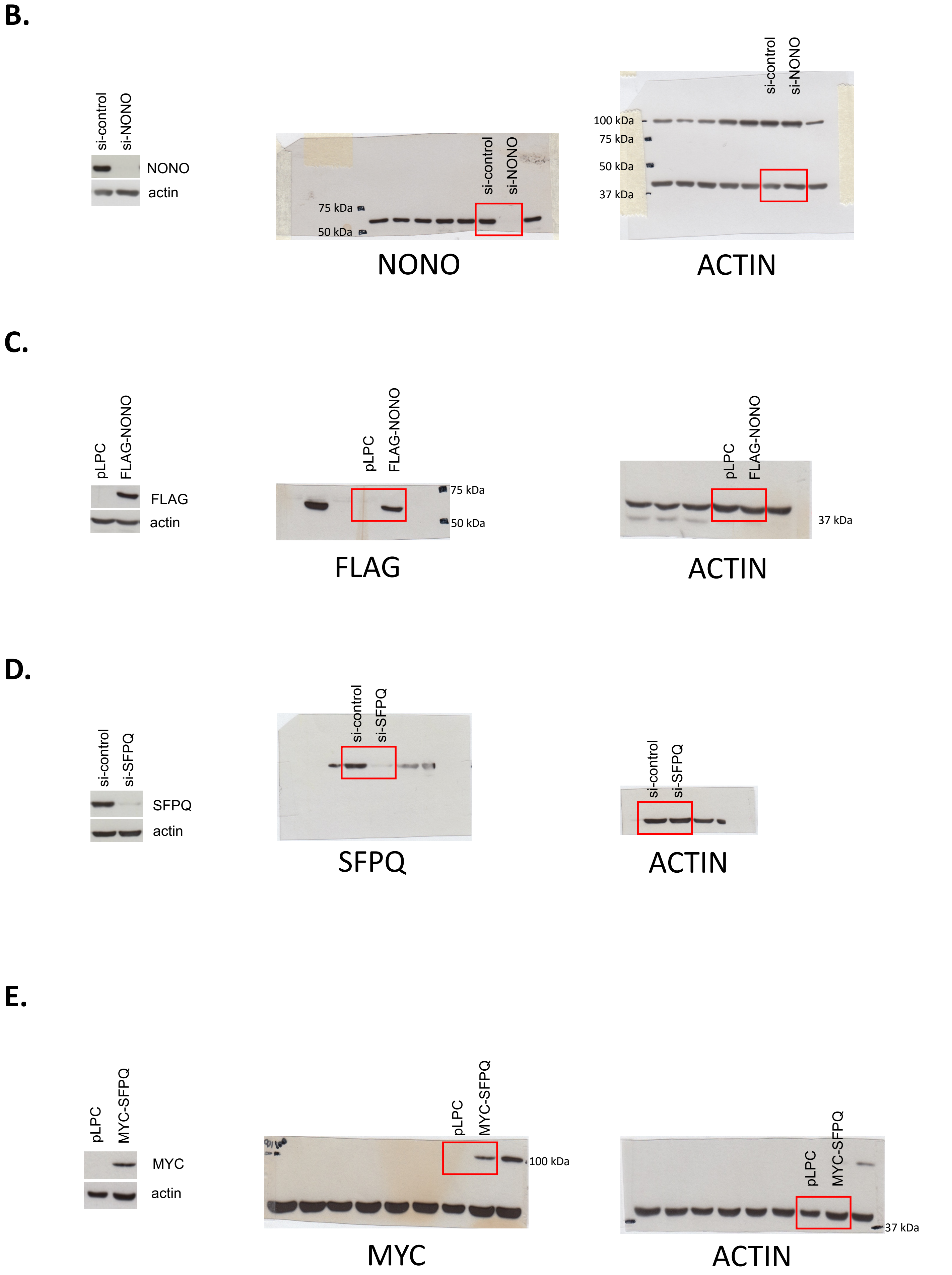

Supplementary Figure 8, referring to western blots in Figure 4
