# Supplementary material for: SFPQ and NONO suppress RNA:DNA-hybrid-related telomere instability
Source: Nat Commun. 2019 Mar 1;10:1001. doi: 10.1038/s41467-019-08863-1 (PMC6397292; doi:10.1038/s41467-019-08863-1)
Supplement: Supplementary file 3 — Reporting Summary [file 41467_2019_8863_MOESM3_ESM.pdf]

nature research

Corresponding author(s): Stefan Schoeftner

Last updated by author(s): 2018/12/07

Reporting Summary

Nature Research wishes to improve the reproducibility of the work that we publish. This form provides structure for consistency and transparency in reporting. For further information on Nature Research policies, see Authors & Referees and the Editorial Policy Checklist.

Statistics

For all statistical analyses, confirm that the following items are present in the figure legend, table legend, main text, or Methods section.

n/a	Confirmed	
☐	☒	The exact sample size (*n*) for each experimental group/condition, given as a discrete number and unit of measurement
☐	☒	A statement on whether measurements were taken from distinct samples or whether the same sample was measured repeatedly
☐	☒	The statistical test(s) used AND whether they are one- or two-sided<br>*Only common tests should be described solely by name; describe more complex techniques in the Methods section.*
☒	☐	A description of all covariates tested
☒	☐	A description of any assumptions or corrections, such as tests of normality and adjustment for multiple comparisons
☐	☒	A full description of the statistical parameters including central tendency (e.g. means) or other basic estimates (e.g. regression coefficient) AND variation (e.g. standard deviation) or associated estimates of uncertainty (e.g. confidence intervals)
☐	☒	For null hypothesis testing, the test statistic (e.g. *F*, *t*, *r*) with confidence intervals, effect sizes, degrees of freedom and *P* value noted<br>*Give P values as exact values whenever suitable.*
☒	☐	For Bayesian analysis, information on the choice of priors and Markov chain Monte Carlo settings
☒	☐	For hierarchical and complex designs, identification of the appropriate level for tests and full reporting of outcomes
☒	☐	Estimates of effect sizes (e.g. Cohen's *d*, Pearson's *r*), indicating how they were calculated

Our web collection on statistics for biologists contains articles on many of the points above.

Software and code

Policy information about availability of computer code

Data collection	Cells subjected to immunofluorescence, RNA FISH, immunofluorescence combined with RNA FISH, CO-FISH or telomere DNA FISH were captured using Leica Application Suite (LAS) imaging software. For confocal acquisition of immunostained cells a EZ-C1 3.91 imaging software (Nikon) was used
Data analysis	The following software was used in this study: ProteinPilotTM software (version 2.0.1; Applied Biosystems) using the ParagonTM algorithm as the search engine for mass spectrometry analysis; ImageJ, Fiji, TFL-TELO and Adobe Photoshop for image analysis and processing; Microsoft Excel and Graph Pad Prism for statistical analysis and graph plotting

For manuscripts utilizing custom algorithms or software that are central to the research but not yet described in published literature, software must be made available to editors/reviewers. We strongly encourage code deposition in a community repository (e.g. GitHub). See the Nature Research guidelines for submitting code & software for further information.

Data

Policy information about availability of data

All manuscripts must include a data availability statement. This statement should provide the following information, where applicable:

- Accession codes, unique identifiers, or web links for publicly available datasets
- A list of figures that have associated raw data
- A description of any restrictions on data availability

The data that support the findings of this study are available from the corresponding author upon reasonable request.

Field-specific reporting

Please select the one below that is the best fit for your research. If you are not sure, read the appropriate sections before making your selection.

☒ Life sciences ☐ Behavioural & social sciences ☐ Ecological, evolutionary & environmental sciences

For a reference copy of the document with all sections, see nature.com/documents/nr-reporting-summary-flat.pdf

Life sciences study design

All studies must disclose on these points even when the disclosure is negative.

Sample size	Sample sizes were chosen according to accepted standards in the field. Sample size was not pre-determined using statistics tolls. As indicated in the figures of the manuscript, minimal size of analyzed biological samples was "3". Statistical analysis (as described in respective figure legends) was used to calculate statistical significance of obtained results. The individual p-values are indicated in all figures.
Data exclusions	No data exclusion
Replication	All experiments were reproducible.
Randomization	No randomization; All experiments are based on gain or loss of function experiments with appropriate controls.
Blinding	No blinding was applied. For critical data, 2 experimenters carried out analysis of biological replicates

Reporting for specific materials, systems and methods

We require information from authors about some types of materials, experimental systems and methods used in many studies. Here, indicate whether each material, system or method listed is relevant to your study. If you are not sure if a list item applies to your research, read the appropriate section before selecting a response.

Materials & experimental systems

n/a	Involved in the study	
☐	☒	Antibodies
☐	☒	Eukaryotic cell lines
☒	☐	Palaeontology
☒	☐	Animals and other organisms
☒	☐	Human research participants
☒	☐	Clinical data

Methods

n/a	Involved in the study	
☒	☐	ChIP-seq
☒	☐	Flow cytometry
☒	☐	MRI-based neuroimaging

Antibodies

Antibodies used

- Mouse anti-Actin, Clone AC-74 (Sigma, A2228)
- Mouse anti-Myc-tag (9B11) (Cell Signaling, 2276)
- Mouse anti-Flag-tag M2 (clone M2) (Sigma, F3165)
- Mouse anti-DNA-RNA Hybrid (S9.6) (Kerafast, ENH001)
- Mouse anti-FUS/TLS (4H11) (Santa Cruz, sc-47711)
- Mouse anti- γH2AX (ser139) (clone JBW301) (Millipore, 06-70)
- Rabbit anti-global histone H3 (Abcam, ab1791)
- Rabbit anti-NONO (Bethyl laboratories, A300-582A)
- Mouse anti-p53 (DO-1) (Santa Cruz, sc-126)
- Rabbit anti-PML (H-238) (Santa Cruz, sc-5621)
- Rabbit anti-PSF/SFPQ (Bethyl laboratories, A301-321A)
- Mouse anti-SFPQ (clone B92) (Sigma, P2860)
- Rabbit anti-phospho RPA32(S33) (Bethyl laboratories, A300-246A)
- Rabbit anti-TRF1(N-19) (Santa Cruz, sc-6165-R)
- Mouse anti-TRF2 (4A794) (Millipore, 05-521)
- Rabbit anti-RAD51 (H-92) (Santa Cruz, sc-8349)
- Rabbit anti-RNase H1 (N2C3) (GeneTex, GTX117624)
- Rabbit phospho-ATR (Ser428) (Cell Signaling, 2853)
- Normal mouse IgG (Santa Cruz, sc-2025)
- Normal rabbit IgG (Santa Cruz, sc-2027)
- Goat anti-rabbit Alexafluor 488 (Invitrogen, A-11008)
- Goat anti-mouse Alexafluor 488 (Invitrogen, A-11001)
- Goat anti-rabbit Alexafluor 555 (Invitrogen, A-21428)
- Goat anti-mouse Alexafluor 555 (Invitrogen, A-21422)

- Goat anti-mouse Alexafluor 647 (Invitrogen, A-21235)
- Goat anti-rabbit IgG HRP-conjugated (Sigma, A6154)
- Goat anti-mouse IgG HRP-conjugated (Sigma, A4416)

Validation	The specificity of rabbit anti-NONO, anti-SFPQ, mouse anti-SFPQ, anti-Flag and anti-Myc was confirmed by western blot analysis of siRNA-depleted cells and cells over-expressing Flag- and Myc-tagged proteins (Fig.4 B-E). The specificity of the rabbit anti-pSer33 RPA32 antibody was confirmed by indirect immunofluorescence staining of cells treated with hydroxyurea (Fig. 3C). Mouse anti-p53 and anti-γH2AX (ser139) antibodies specificity was tested by western blot experiments performed on cells treated with hydroxyurea (Supp. Fig. I, J). The specificity of mouse anti-TRF2 antibody was confirmed by chromatin immunoprecipitation experiment, showing a specific interaction with telomeric repeats but not with AluY repeats (Fig. 1G). Rabbit anti-RNase H1 and anti-TRF1 antibodies specificity was validated by indirect immunofluorescence staining of siRNA-depleted cells (Suppl. Fig. 2 K-M). The specificity of the anti-RNA:DNA hybrids S9.6 antibody was confirmed by indirect immunofluorescence staining performed in cells depleted for RNase H1, used as positive control (Fig. 2 F, H). Rabbit anti-phospho-ATR antibody specificity has been stated on the manufacturer's website (Cell Signalling technology). The specificity of rabbit anti-PML was previously validated by Ahmed, A. et al. "Regulation of NF-κB by PML and PML-RARα." (Sci. Rep. 7, 44539; doi: 10.1038/srep44539 (2017)).<br>Mouse anti-FUS/TLS antibody specificity was previously validated by Zhang, T. et al. "FUS regulates activity of microRNA-mediated gene silencing." (Molecular Cell. doi:10.1016/j.molcel.2018.02.001 (2018)).<br>The specificity of rabbit anti-RAD51 antibody was previously validated by Serrano, M.A. et al. "DNA-PK, ATM and ATR collaboratively regulate p53-RPA interaction to facilitate homologous recombination DNA repair." (Oncogene. 2013 May 9; 32(19): 2452–2462. doi:10.1038/onc.2012.257). Normal mouse IgG and normal rabbit IgG were used as negative control as shown in Fig. 1G, Supp. Fig. 1A-D.

Eukaryotic cell lines

Policy information about cell lines

Cell line source(s)	U2OS and H1299 cells were obtained by ATCC
Authentication	Cells were obtained by ATCC; characteristic features such as telomere length, telomere length distribution, the presence of APBs and metaphase chromosome characteristics was experimentally validated
Mycoplasma contamination	All cell lines used in the study were tested negative for mycoplasma contamination
Commonly misidentified lines (See ICLAC register)	not applicable
